# Use of Electronic Noses in Seawater Quality Monitoring: A Systematic Review

**DOI:** 10.3390/bios8040115

**Published:** 2018-11-23

**Authors:** Alessandro Tonacci, Francesco Sansone, Raffaele Conte, Claudio Domenici

**Affiliations:** National Research Council of Italy, Institute of Clinical Physiology (CNR-IFC), Via Moruzzi 1, 56124 Pisa, Italy; francesco.sansone@ifc.cnr.it (F.S.); raffaele.conte@ifc.cnr.it (R.C.); domenici@ifc.cnr.it (C.D.)

**Keywords:** chemical sensors, eNose, environmental monitoring, seawater, sensors, volatile organic compounds

## Abstract

Electronic nose (eNose) systems are particularly appreciated for their portability, usability, relative low cost, and real-time or near real-time response. Their application finds space in several domains, including environmental monitoring. Within this field, marine monitoring is of particular scientific relevance due to the fragility of this specific environment, daily threatened by human activities that can potentially bring to catastrophic and irreversible consequences on marine wildlife. Under such considerations, a systematic review, complying with the PRISMA guidelines, was conducted covering the period up to 15 October 2018, in PubMed, ScienceDirect, and Google Scholar. Despite the relatively low number of articles published on this specific topic and the heterogeneity of the technological approaches employed, the results obtained by the various groups highlight the positive contribution eNose has given and can provide in near future for the monitoring and safeguarding of this delicate environment.

## 1. Introduction

Anthropogenic activities produce every day large quantities of pollutants that are discharged in the environment [1,2], including oceans, and in the seawater at large. Such discharges, together with the exploitation of the sea resources nowadays represent a significant threat for the marine environment and cause a continuous, unprecedented degradation of oceans, seas and coastal areas [3,4,5]. The monitoring of such compounds is a pivotal action when aiming at promoting the preservation of marine and coastal areas and the sustainability of the ecosystem, at large [6].

To this extent, the approaches commonly used are based on traditional water and air quality evaluation methods, including analytical tools that, however, commonly operate at laboratory settings, and are therefore (i) unable to provide real-time or near real-time results, and (ii) possibly being associated with the occurrence of sample degradation during transportation from the sampling site to the laboratory bench.

To properly overtake such bottlenecks, portable devices can represent a smart, mostly robust, easy-to-use, low-cost alternative, capable of bringing fast, reliable, reproducible responses, without the need for sample transportation and, likely, consequent degradation.

Among such systems, electronic nose (eNose)-like tools are emerging as a popular alternative, maximizing the above mentioned advantages and keeping, at the same time, low drawbacks.

The first evidences for eNose systems dates back to the early 1980s, when Persaud and Dodd [7] first attempted mimicking the functioning of the mammalian olfactory system by means of an “electronic nose”. Since then, a plethora of systems has been realized for several different applications, including biomedical and diagnostic, environmental, as well as food industry/food quality usage [8,9].

Under such considerations, here we present a systematic review to investigate the use of eNose systems within a specific field of application, represented by the monitoring of seawater pollution. To this extent, we will first outline the search strategy adopted for this work, then we will present the results obtained, critically discussing such evidences, and highlighting pros and cons of the single approaches.

## 2. Materials and Methods

### Search Strategy

**Figure 1:**
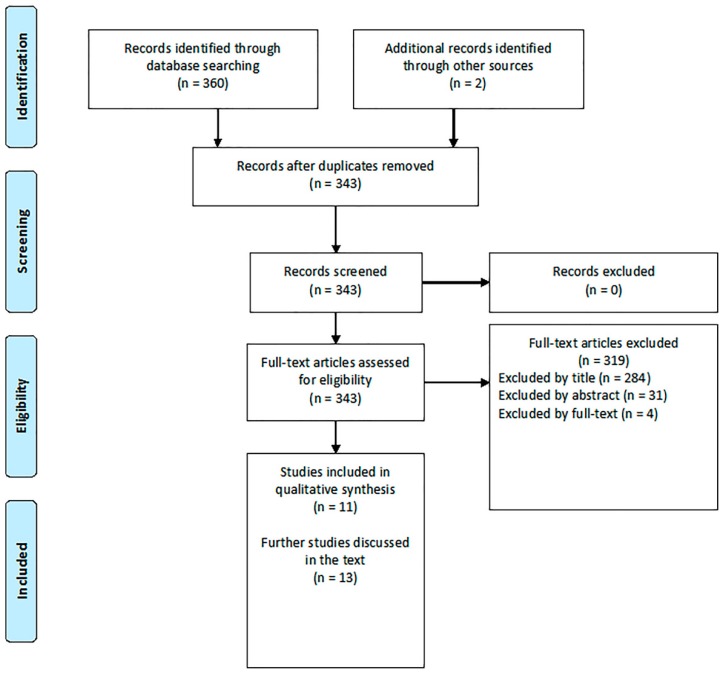
Flowchart for literature review.

A systematic review of the literature, covering the period up to 15 October 2018, was conducted in PubMed, ScienceDirect, and Google Scholar database, according to the PRISMA guidelines [10]. The search strategy was as follows: (“eNose” or “electronic nose”) and (“seawater pollution” or “seawater monitoring” or “seawater” or “pollution”). The search was limited to research articles published in English language in peer-reviewed journals or in international conferences’ proceedings. After having discarded multiple hits, the results obtained were sorted by relevance and the most significant works dealing with seawater monitoring by means of eNose systems were selected (as displayed in Figure 1). Given the relatively low number of systems specifically designed for seawater monitoring, some tools designed for water monitoring in general, but adaptable to marine monitoring, were also included and critically discussed.

## 3. Results

This systematic review retrieved a handful articles directly dealing with seawater monitoring by eNose systems. The articles taken into account are displayed in Table 1.

Specifically focusing on the results reported in Table 1, one of the first studies performed in this field was conducted by Bourgeois and colleagues [11] in the UK. The work described the development and use of an eNose system for monitoring water and wastewater, potentially usable also for seawater assessment. The first prototype was based on an eNose5000 system consisting of an array of 12 conducting polymers, interfaced with a sampling vessel and with a PC for data analysis. Aside from this laboratory prototype, a second system was realized, with a ProSAT sensor array with eight conducting polymers as the sensing system, a PC for data storage and a data transfer system. This second system was tested on-field at the Cranfield University Sewage Works facilities and, through a proper conditioning in a temperature-controlling environment, has been demonstrated to be able to reduce humidity variations, at the same time improving reproducibility. The system was capable of detecting the presence of low concentrations (around 5 ppm) of 2-chlorophenol, with optimal performances at 25.5 °C of temperature, 170 mL/min of flow, and 0 of porosity.

As widely known, hydrocarbons are among the major pollutants of the marine environment, with oil spills occurring throughout the seas and the oceans as a consequence of marine accidents, petroleum pipeline leakages and fraudulent events. Therefore, systems capable of specifically assessing the presence of oil spills on the sea surface can be critical to reduce the environmental impact of such discharges in marine and coastal environment.

Under such consideration, Tzing and colleagues [12] developed a system, based on the eNose technology, to identify the source of spilled oil in an accident site, comparing it with a “gold standard” system composed by a gas chromatography—mass spectrometry (GC-MS) device that served to ensure the correct classification of the source site.

The system developed was composed of a Cyranose 320 eNose system, composed of an array of 32 polymer sensors, a pump for air inlet and purge, as well as a PC equipped with a dedicated software for data acquisition and analysis. Together with this system, a so-called z-Nose system was employed, constituted by an Electronic Sensor Technology FGC/SAW 7100 eNose, equipped with a pre-concentrating trap containing Tenax absorbent, a short gas chromatography (GC) column, a pneumatic control, a fast SAW detector, as well as a programmable gate array microprocessor. As for reference analysis, a Varian CP-3800 GC connected to a Saturn 2000 ion-trap mass spectrometer (MS) was used.

Spilled oil samples were collected from surface water at the site of an accident, whereas a set of known compounds, including gasoline, jet fuel, diesel, and fuel oil were also collected, properly stored and transported to the laboratory, representing the reference samples for the eNose (1 mL of oil loaded into a 30 mL vial, equilibrated at ambient condition for 5 min).

By means of a PCA, the eNose classified the unknown stimulus as “jet fuel”, as further agreed by zNose and GC-MS systems, demonstrating the good classification capability of the eNose system. However, the main limitation of this promising approach resides in the fact that the test set employed was represented by only one sample that, despite the high internal consistency of the eNose response, could lead to misleading conclusions.

A couple of years later, Goschnik et al. [13] employed the semiconductor-based eNose system KAMINA to analyze water samples polluted with chloroform (chloro-organic solvents-like) or ammonia (fecal contamination-like). Despite this specific trial was mainly dealing with wastewaters, this application could have had the potentialities to be translated into a system usable for seawater monitoring, therefore, it was added to the present investigation. The trials were performed with a flow rate of 500 mL/min and an entrance temperature of 20 °C, subsequently increased up to 240 °C and 300 °C at the two microarray ends, respectively. The results obtained for this investigation, showing a detection limit below 1 ppm, demonstrated the usefulness of the KAMINA system for this purpose, however revealing a strong need for building up a populated, reliable reference database to support the correct classification by the eNose system. The absence of such a support would dramatically decrease the performances of the system, as frequently occurs with eNose approaches.

A very interesting approach was proposed by Lozano et al. in 2014 [14] and updated a few years later [15]. There, the authors proposed a portable eNose system equipped with an IEEE 802.11 transceiver for wireless communication in order to be ready for inclusion within a wide eNose network for distributed measurements. The system developed, also featuring an electronic pump and valve, as well as embedded electronics and rechargeable batteries, was able to work with several different resistive microsensors. By performing measurements on cycles of 60 s of adsorption and 540 s of desorption by means of an headspace sampling system, the prototype presented was demonstrated to be able to reliably discriminate between pollutants (especially when a relatively low number of compounds are taken into account) using principal component analysis (PCA) and a few different artificial neural networks (ANNs). More specifically, the compounds used to evaluate the response of the system included: blank water, acetone, toluene, ammonia, formaldehyde, hydrogen peroxide, ethanol, benzene, dichloromethane, acetic acid, xylene and dimethylacetamide. The correct discrimination occurred in more than 90% of samples in most cases, with concentrations of the various compounds of around 1 mL in a 20 mL vial at 16 °C constant temperature.

Specifically dealing with seawater pollution, the work published by Tonacci and colleagues [16] described the implementation of an eNose system to be used within an autonomous underwater vehicle (AUV) for monitoring oil leakages in a marine protected area, including the Tuscan Archipelago and Cetacean Sanctuary, North Mediterranean. The system implemented was composed by a sensing part, featuring Photo-Ionization Detectors (piD), a system for air inlet and purge made up of an electronic pump and a valve, and a smart embedded electronics, based on an Arduino Mega 2560 board. A PC for data acquisition was also foreseen, and ANNs of the type Kohonen self-organizing map (KSOM) were implemented and trained in order to: (i) recognize the level of pollution, independently from its source (e.g., high, medium, low level), and (ii) identify the substance detected within a set of known compounds (e.g., crude oil, diesel fuel, gasoline, jet fuel). Despite the good accuracy of classifiers, especially for the first KSOM (around 74% of correct classification for the first KSOM, 67% for the second one, and a fast response (below 20 s), with pollutants’ concentration of 10^−3^ vol/vol), the system was seen to provide not reliable response when exposed to relative humidity (RH) above 70%, thus representing a noteworthy limitation of the tool described. Two further expansions of this application were also presented by the same group. The first one adopted the same approach including the eNose system within a moored buoy in order to combine dynamic (AUV) and static (buoy) monitoring of a given marine area [19], whereas the second one scaled the payload system in order to make room for future inclusion of such system within a real-time context of distributed monitoring [17].

A novel approach to the problem was adopted by Son and colleagues in 2015 [18]. They developed a bio-eNose consisting of human olfactory receptors, subcloned in pcDNA3 mammalian expression vectors containing the first 20 amino acids of human rhodopsin (Rho-tag), and single-walled carbon nanotube field-effect transistor (swCNT-FET). The system functioning was assessed to distinguish the presence of geosmin and 2-methylisoborneol, compounds produced by bacteria and reliable indicators of contamination in the water supply system. The approach adopted can be tailored upon the users’ needs also to investigate the presence of contaminants in seawater. The system was demonstrated to be reliable in detecting the proposed stimuli at concentration as low as 10 ng/L, with the possibility to assess the presence of both compounds at the same time, thanks to the specific binding between hOR51S1 and hOR3A4 and geosmin and 2-methylisoborneol, respectively. Very importantly, the correct classification of the eNose system does not require a specific pre-treatment of water samples, allowing the use of this solution for rapid monitoring of water quality. The binding between other olfactory receptors and specific pollutants could enable applying the solution to other, customized applications and experimental settings.

Recent technological advances allowed researchers to explore new solutions in this specific field. Internet of Things (IoT), for example, represents a landmark revolution in the field of sensing and bio-sensing. This promising and extremely actual approach was employed by Climent and colleagues [20], which developed a low-cost, portable eNose, named Multisensory Odor Olfactory System (MOOSY4), for water quality assessment purposes. In that work, the MOOSY4 was employed for the quality control of bottled water, but can be also used for seawater monitoring. The prototype was composed of four metal oxide semiconductor (MOS) gas sensors, suitable for the IoT technology, with a system architecture featuring data acquisition, storage, processing and user interfacing parts. The volatiles monitored, dimethyl disulphide, dimethyl diselenide, sulphur, were correctly recognized in 86% of cases, making the system extremely suitable for the purpose it was conceived for, and potentially applicable to a wide range of volatiles monitoring.

As hydrocarbons are, as reported, the main source of marine environmental pollution, specific approaches have been recently adopted tailored at this peculiar domain. For example, Aliaño-González and colleagues [21] used an AlphaMOS eNose combined with chemometrics to identify and discriminate 444 samples composed 40 µL of different petroleum-derived products (PDPs), including gasoline, diesel and paraffin, poured on a support and subjected to a natural weathering process by evaporation for one month. Taking advantage of the use of the linear discriminant analysis allowed the scientists to perform a correct discrimination of presence/absence of PDP in all cases and of the different PDPs in 97.7% of samples.

## 4. Discussion

The problem of environmental pollution is central in today’s society, and full attention is paid by both National and International Environmental Organisms, as well as researchers, in finding solutions that can properly respond to the pressing necessity of environmental safeguarding. The extremely fragile ecosystem represented by marine and coastal areas is continuously threatened by manmade activities (e.g., ship transits, industrial discharges, etc.) that, in the medium- to long-term, can result in serious and irreversible consequences for marine areas and their wildlife [22,23].

To this extent, several initiatives, including national and international research projects, task-forces, position papers, and regulations have been promoted in last decades, many of which highlighting the need for tailored interventions both in a more preventive sense (monitoring of oil spills, etc.) and concerning accident remediation [24]. On the edge between these two phases, a number of smart monitoring systems have been developed in many research centers, to ensure a proper monitoring of a given area and, at the same time, allowing the possibility to trigger eventual alarms when the concentration of pollutants or, more specifically, of a single pollutant, exceeds a given safety threshold. Such solutions are especially useful in marine protected areas, where even the detection of very low levels of pollutants could enable the competent authorities to undergo quick, effective countermeasures, often preventing real environmental disasters. Within this framework, the use of low-cost, sensitive tools, ensuring a fast, real-time, or near real-time response is of particular interest, and this fact paved the way for the employment of eNose systems for this purpose.

In the last years, several prototypes and products based on this technology have been developed and employed for seawater monitoring, using different technologies as time flowed.

Among the most widely used technologies, especially in the first works published, are conducting polymers [11,12], providing good results, but in some cases obtained on a small number of trials or compounds. However, it is worth noting that such sensors are largely affected by environmental conditions, that could dramatically impact on the recognition performances of the tool, overall. The experimental setting is also pivotal with this approach, since the sensors should often be carefully cleaned after each measurement, reducing their applicability in a real environmental setting for continuous measurements unless a coupled purging device is used.

Semiconductors, which are the basis of most eNose systems developed to date, were also employed several times to this extent [13,20,21] and, considering the timeline of existing publications on the argument, are probably the most actual approach to the problem. Some contrasting results are present under this technology (e.g., considering the limits highlighted by the work by Goschnik and colleagues), however, the use of such devices probably guarantees the best performances, to date. The main limitation identified under this approach is the need for a well populated reference database to train the related recognition algorithms, but with the technological developments of recent times the issue was, in most cases, successfully resolved.

Other approaches retrieved in this domain included the use of photo-ionization detectors [16,17,19], featuring very fast response times, however displaying significant issues with high humidity environments and being characterized by high costs, and resistive detectors like the ones employed by Lozano and colleagues [14] and Herrero and colleagues [15]. The last solution mentioned is characterized by the strong need to control environmental conditions for the signal stability, a common problem with most of the other technologies normally employed in this specific field.

Additionally, promising hybrid approaches including a biological part, consisting of human olfactory receptors, as well as a technological part, were successfully employed by Son and colleagues [18], possibly opening the way for new explorations to reduce the impact of one of the most known issues of the eNose systems, that is non-specificity. In fact, thanks to the selective bond between compound and receptor, this approach could enable the detection of specific compounds, even though the reversibility of this bond remains questionable. The provision of such devices for new, promising technologies, including the IoT, as already experimented by Climent et al. [20], could enable new perspectives, at large in environmental, and specifically in seawater, monitoring.

## Figures and Tables

**Table 1:** Study selected (AUV: autonomous underwater vehicle; FGC: fast gas chromatography; GC-MS: gas chromatography―mass spectrometry; GSM: geosmin; IoT: Internet of Things; MIB: 2-methylisoborneol; MOS: metal oxide semiconductor; ORs: olfactory receptors; piD: photo-ionization detector(s); swCNT-FET: single-walled carbon nanotube field-effect transistor).

Study	Design	Sensor(s) Type	Monitoring Site
Bourgeois et al. [11]	(i) Sampling vessel, eNose sensor array and PC for data analysis; (ii) eNose sensor array, PC for data storage, data transfer system	(i) eNose5000; (ii) ProSAT	(i) Laboratory trials; (ii) Cranfield University Sewage Works facilities
Tzing et al. [12]	eNose used to identify source of oil leakage from an accident site. zNose, GC-MS used for results’ confirmation and quantitative analysis	eNose: Cyranose 320; zNose FGC/SAW 7100; GC-MS: Varian CP-3800 + Saturn 200 ion-trap	On-field (accident site)
Goschnik et al. [13]	eNose used to discriminate between clear and polluted (chloroform and ammonia) water	Semiconductor-based KAMINA eNose	Laboratory trials
Lozano et al. [14,15]	Portable wireless resistive sensor-based eNose, electronic pump and valve, electronics and rechargeable batteries. Measurements on clear water and 11 pollutants	Home-made eNose capable of hosting resistive sensors	Laboratory trials
Tonacci et al. [16,17]	eNose, electronic pump and valve, embedded electronics and PC for data acquisition, integrated within an AUV	eNose composed of three piD sensors	On-field acquisition (La Spezia Gulf and Enfola Bay, Italy)
Son et al. [18]	bio-eNose tested to distinguish the presence of GSM and MIB	Human ORs and swCNT-FET	Laboratory trials with tap water, bottled water and river water
Moroni et al. [19]	eNose, electronic pump and valve, embedded electronics and PC for data acquisition, integrated within a moored buoy	eNose composed of three piD sensors	On-field acquisition (La Spezia Gulf and Enfola Bay, Italy)
Climent et al. [20]	eNose, architecture for data acquisition, storage, processing and user interfacing parts	eNose composed of four, IoT suitable, MOS sensors	Laboratory trials with water polluted with dimethyl disulphide, dimethyl diselenide, sulphur
Aliaño-González et al. [21]	eNose combined with chemometrics; linear discrimination analysis for classification	AlphaMOS eNose composed of MOS sensors	Laboratory trials on 444 samples from gasoline, diesel and paraffin, subjected to a natural weathering process by evaporation
